# Understanding Advance Care Planning in oncology: Barriers, perceptions, and pathways toward patient-centered decision-making

**DOI:** 10.1017/S1478951526101655

**Published:** 2026-02-06

**Authors:** Fulvio Bergamo Trevizan, Carlos Eduardo Paiva, Livia Costa de Oliveira, Karla Santos da Costa Rosa, Bianca Sakamoto Ribeiro Paiva

**Affiliations:** 1 Research Group on Palliative Care and Health-Related Quality of Life (GPQual), Barretos Cancer Hospital, Barretos (SP), Brazil; 2 Institute of Education and Research, Barretos Cancer Hospital, Barretos (SP), Brazil; 3 Department of Clinical Oncology, Breast and Gynecology Division, Barretos Cancer Hospital, Barretos (SP), Brazil; 4 Palliative Care Unit, National Cancer Institute, Rio de Janeiro, Brazil

**Keywords:** Advance Care Planning, palliative care, barriers, cancer, decision-making

## Abstract

**Objectives:**

To explore cancer patients’ understanding of Advance Care Planning (ACP) and identify the main barriers hindering its effective implementation in clinical practice.

**Methods:**

This qualitative descriptive study included Brazilian women with breast cancer aged 18–75 years, all with preserved functional status, recruited by convenience sampling. Exclusion criteria were difficulty using online calls or significant communication impairment. Data collection involved a sociodemographic questionnaire and a follow-up interview. After receiving an informational brochure, participants were contacted by video call 14 days later and asked, “How do you understand what ACP is?” Interviews were conducted confidentially at home, transcribed, and analyzed according to qualitative research reporting guidelines.

**Results:**

Sixty-one women participated. Most had difficulty understanding ACP; nearly 40% could not define it. Main barriers included cultural resistance to discussing death, reliance on family members or physicians for decision-making, and lack of clear information. Many participants confused ACP with preventive care. A conceptual multilevel model was developed, showing how cultural taboos, family dependence, and systemic inertia interact to sustain barriers through a feedback loop in which cultural avoidance reinforces structural gaps and institutional neglect.

**Significance of results:**

This study provides evidence on how ACP is understood and misinterpreted by cancer patients in a middle-income Latin American setting, an area that remains underrepresented in the literature. By demonstrating that misconceptions, cultural taboos, and systemic barriers operate through a reinforcing multilevel process, the findings offer a conceptual framework that explains why ACP remains marginal in routine oncology care. The model highlights critical points for intervention, including patient education, professional communication, and institutional support, and is directly applicable to similar sociocultural contexts characterized by strong family involvement and biomedical dominance. These results have clear implications, supporting the integration of ACP as a proactive, relational, and value-based process rather than a late end-of-life intervention.

## Introduction

Advance Care Planning (ACP) enables patients to express their healthcare preferences and ensures that their values guide medical decisions, particularly in situations of incapacity or end-of-life (EoL; Sudore et al. 2017; Mori et al. 2025). Despite its recognized benefits such as reducing anxiety, improving quality of death, and preventing unnecessary aggressive interventions, ACP remains underutilized in Brazil (Rocha Tardelli et al. 2023). Evidence suggests that limited adoption is not only solely attributable to structural or systemic barriers but also reflects significant gaps in knowledge, cultural misconceptions, and discomfort surrounding EoL discussions among patients, families, and healthcare professionals (Guccione et al. 2023; Pimsen et al. 2025).

While several countries have incorporated ACP into national healthcare policies, promoting communication, shared decision-making, and respect for patient autonomy (Yennurajalingam et al. 2013; Borenko et al. 2023), Brazil remains considerably behind in this regard (Rocha Tardelli et al. 2023; Trevizan et al. 2024). The absence of structured ACP initiatives perpetuates the stigma around EoL discussions, reinforces medical paternalism, and restricts patient autonomy (Goswami 2023; Zabawa 2023). Additionally, cultural and institutional barriers sustain resistance to ACP, leading to reactive rather than proactive healthcare decisions (Dias et al. 2022).

Although ACP has been globally recognized as a key component of high-quality care, its implementation in low- and middle-income countries, particularly in Latin America, remains limited and poorly documented. Existing evidence indicates that only a minority of Latin American nations have formal ACP regulations, and where these exist, they often reproduce legalistic, North American-inspired models that fail to reflect the region’s sociocultural and relational contexts (Soto-Perez-de-Celis et al. 2016; Tardelli et al. 2025). The insufficient availability of educational initiatives and institutional guidelines adapted to local healthcare systems underscores the urgent need for context-specific strategies (Cajavilca et al. 2024; Tardelli et al. 2025).

Regional analyses show that none of the countries in the region provide adequate ACP training for health professionals, and that many clinicians report legal uncertainty when engaging in EoL conversations (Tardelli et al. 2025). These challenges extend beyond regulatory limitations, reflecting systemic gaps such as the absence of ACP in health curricula, insufficient communication training, and the limited integration of Palliative Care (PC) into national health systems. Addressing these barriers requires not only descriptive inquiry but also the development of feasible, evidence-informed strategies that support ACP implementation in real-world clinical contexts. However, how ACP is interpreted, operationalized, and integrated into oncological care in Brazil and other Latin American countries remains poorly understood, as few empirical studies have examined the educational, operational, and cultural determinants of its adoption (Trevizan et al. 2024, 2023; Cajavilca et al. 2024; Álvarez Acuña et al. 2025; Tardelli et al. 2025; Arias-Rojas et al. 2025a).

Beyond these structural barriers, the paucity of research on ACP in Latin America also reflects broader cultural characteristics of region’s healthcare and educational systems. The dominance of a curative biomedical model, fragmented care pathways, and the lack of early EoL discussions contribute to the scarcity of ACP-related studies (Trevizan et al. 2024; Tardelli et al. 2025). Therefore, research in this field should prioritize the identification of feasible and culturally sensitive approaches to expand ACP implementation and strengthen patient-centered care.

International debates have underscored the importance of reframing ACP as a dynamic, relational, and socially embedded process rather than a purely medical intervention. Emerging evidence highlights that patients’ and caregivers’ engagement in ACP is influenced by emotional readiness, family dynamics, and cultural meanings surrounding illness and autonomy. Consequently, future research should move beyond distal clinical outcomes, such as goal-concordant care or documentation rates, and explore the experiential dimensions of ACP, including feelings of safety, mutual understanding, strengthened relationships, and reduced caregiver burden (De Vleminck and den Block L 2023).

Given this context, the present study seeks to deepen understanding of the barriers and facilitators of ACP in Brazil and to generate evidence that can inform educational, clinical, and policy initiatives across Latin America. By exploring cancer patients’ understanding of ACP, this study aims to explore cancer patients’ understanding of ACP and identify the main barriers that hinder its implementation, preventing the effective integration of ACP into routine oncology care.

## Methods

### Design study

Qualitative descriptive study, constituting the second phase of a larger investigation, building upon previously published quantitative data (Trevizan et al. 2024).

### Participants

Patients were recruited by convenience sampling from the Women’s Outpatient Clinic and the Chemotherapy Infusion Center of a large Brazilian cancer hospital, recognized as one of the main reference centers for oncology care in Latin America. These settings provided access to women in different stages of treatment and follow-up, allowing for a diverse representation of experiences. Eligible participants were Brazilian women aged 18–75 years, with a confirmed diagnosis of breast cancer and an Eastern Cooperative Oncology Group Performance Status (ECOG-PS) ≤ 2. Patients were excluded if they had difficulty participating in online interviews or presented significant auditory, visual, or verbal communication impairments that could hinder the dialogue process.

### Data collection and analysis

Initially, patients completed a sociodemographic questionnaire and received an informational brochure providing concise information about the concept of ACP. After 14 days, they were contacted via video conference and asked the question: *How do you understand what* ACP *is?* They were instructed to describe their understanding of the concept. The data collection took place while the patients were at home, alone, in a safe environment, with confidentiality guaranteed. Each interview lasted an average of 20 minutes.

Interviews were conducted by a male psychologist (PhD) experienced in qualitative methods and psycho-oncology. The researcher had formal training in qualitative interviewing and previous experience conducting studies in PC and ACP. He had no prior therapeutic or hierarchical relationship with participants, minimizing potential power asymmetries. Participants were informed that the interviewer was a researcher studying PC and ACP. Reflexive notes were written after each interview to document contextual impressions, non-verbal cues, and emerging analytical issues.

The interviews were recorded and transcribed in Portuguese, verified for accuracy by three independent researchers, and analyzed using Bardin’s method, following the Consolidated Criteria for Qualitative Research Reports (Tong et al. 2007). Manual coding was conducted without software assistance. Codes and categories were developed inductively from the data, and thematic convergence was discussed until consensus was reached. Discrepancies were resolved through analytical meetings to ensure reliability. Participants did not review transcripts or final interpretations. Representative quotations were selected to illustrate each theme in the results section. This study was approved by the Research Ethics Committee (approval number 4,987,629) on September 2021. All participants provided informed consent before participation.

## Results

The study included 61 women, primarily married (78.7%, *n* = 48), Catholic (60.7%, *n* = 37), non-working (56.5%, *n* = 34), and with a family income of 1–3 minimum wages (54.8%, *n* = 33). Most had completed high school (47.5%, *n* = 29). Metastases were present in 47.5% (*n* = 29), and stage IV cancer in 45.9% (*n* = 28). Palliative systemic treatment was predominant (59.0%, *n* = 36).

Participants showed significant difficulty understanding ACP, about 40% (*n* = 25) were unfamiliar with the term or reluctant to discuss EoL issues. Around 35% (*n* = 21) confused ACP with general health care and prevention, without distinguishing it from EoL decision-making. Only 25% (*n* = 15) correctly identified ACP as a tool for self-determination and planning healthcare decisions in situations of incapacity or near EoL.

Each interview transcript was analyzed semantically to assess the participants’ level of understanding of ACP. Responses were categorized as correct understanding, when ACP was recognized as a process of shared decision-making and autonomy regarding future care; partial understanding, when it was misconceived as general health maintenance or preventive care; and unfamiliarity, when participants demonstrated limited or no knowledge of the concept. Table 1 presents a comparative synthesis of these findings.

**Table 1.** Comparative synthesis of participants’ discourses on Advance Care Planning according to level of understanding and thematic dimensions

Thematic dimensions	Correctly understanding	Confused	Unfamiliar or reluctant
**Meaning/definition of ACP**	*“It’s a way to organize everything as you wish, so people understand your preferences.”* [P. 1]	*“It’s about prevention, doing exams, taking care of yourself before getting sick.”* [P. 4]	*“I don’t know what it is.”* [P. 20]
	*“It’s about deciding who will speak for me when I can’t make decisions.”* [P. 45]	*“It’s when doctors try to solve a health problem before starting treatment.”* [P. 11]	Did not respond. [P. 50]
**When ACP should be discussed**	*“At the beginning of treatment, because everything is easier to explain.”* [P. 17]	*“Whenever the patient feels ready, it depends on each person.”* [P. 10]	*“When the patient is very sick, with tubes, almost at the end.”* [P. 22]
**How ACP should be discussed**	*“It should be through the medical team, then discussed with the family, but my opinion should prevail.”* [P. 5]	*“Personally is better, but it could also be by video call.”* [P. 9]	*“It’s hard to tell someone they are going to die; it’s uncomfortable.”* [P. 29]
**Setting/mode of the conversation**	*“During a medical consultation, when there’s time to talk properly.”* [P. 13]	*“Through online meetings or informative booklets.”* [P. 25]	*“It could be in a group, after treatment sessions.”* [P. 20]
**Tone and communication style**	*“Direct and honest, but comforting.”* [P. 18]	*“It must be gentle, not scaring the patient.”* [P. 14]	*“Better not to say it directly; people get scared.”* [P. 27]
**Role of the family**	*“The family should be together, because they will be the ones caring.”* [P. 8]	*“The decision is mine, but they should listen.”* [P. 15]	“Better without them, they get too emotional.” [P. 24]
**Barriers mentioned**	*“Families avoid talking about it, they think it means giving up.”* [P. 30]	*“People get scared when you talk about death.”* [P. 7]	*“Doctors don’t have time to explain everything.”* [P. 26]

*Notes*: *ACP: Advance Care Planning; P: Participant ID.*

The comparative analysis revealed heterogeneous levels of understanding of ACP among participants. While some demonstrated a clear perception of ACP as a means to preserve autonomy, document preferences, and anticipate future care decisions, others interpreted it merely as a form of preventive or routine health management. A subset of participants expressed complete unfamiliarity with the concept. Beyond these conceptual variations, the findings underscore emotional and cultural barriers, such as fear of discussing death and misconceptions that associate ACP exclusively with terminality (Table 2). Participants who exhibited a more accurate understanding of ACP tended to emphasize its relational dimension, highlighting dialogue, family involvement, and professional empathy. In contrast, those with partial or absent understanding often relied on a predominantly biomedical or procedural perspective.

**Table 2.** Barriers to Advance Care Planning identified through qualitative analysis: domains, themes, and participant quotations

**Domain 1. Cultural and Psychosocial Barriers**	
Analytical Categories	Representatives **quotations**
Cultural resistance to discussing death and dying	*“It’s complicated. How do you approach someone and tell them they are going to die? Or that someone has died? It’s uncomfortable, but sometimes it’s necessary, and it can’t be avoided.” [P. 28]*
Denial of advanced illness or the possibility of death	*“It’s about exchanging ideas to see if it’s possible to find another type of treatment, not just palliative care, but maybe a cure.” [P. 80]*
Fear of causing emotional distress when addressing EoL issues	*“[…] Preparing the patient emotionally for the end. […] Helping us accept that the end will come, so we don’t suffer as much.” [P. 93]*
Sociocultural patterns of delegated decision-making	*“[…] With the family involved, for sure. It’s the family who will take care, think and reflect on what the patient needs. The family needs to be aware and informed.” [P. 139]*
Cultural norms that value silence about death and avoid difficult conversations	*“[…] I’m not sure how to answer, because I think that, now, I don’t need ACP. I don’t need it because, thank God, everything is going well. […] Right now, I’m not very concerned about it. I don’t know if I’ll change my mind in the future.” [P. 65]*

**Domain 2. Cognitive and Conceptual Barriers**	
Analytical Categories	Representatives **quotations**
Conceptual confusion between ACP and preventive care	*“ACP would it be a way to plan my care before the illness? Is it prevention? The word ‘prevention’ comes to mind. […] Prevention before getting sick and during the illness, where you treat yourself to avoid worsening, to get better.” [P. 18]*
Low health literacy regarding future care planning	*“From what I understood, it means having regular check-ups. Seeing a doctor a few times to find out if, in the future, you might develop a disease and to detect it early.” [P. 21]*
Lack of knowledge about ACP	*“I wouldn’t say I know completely, but what I think it means anticipating. Let’s suppose […] I have surgery tomorrow, and the doctor tells me to take precautions: ‘J., today you need to do this, this, and this so that you’ll be ready for tomorrow.’ So, I anticipated it. […] I prepare myself a day in advance.” [P. 34]*
Difficulty understanding autonomy and advance decision-making concepts	*“[…] I couldn’t really absorb the information. […] The patient needs to be aware of everything, gathering information to bring clarity.” [P. 30]*

**Domain 3. Structural and systemic barriers**	
Analytical Categories	Representatives **quotations**
Lack of access to clear and accessible information about ACP	*“The doctor can come and talk to the patient, but they need to explain from the beginning, so the person is aware of what is happening and what may happen. Talking with the doctor, getting guidance, is much better. […] Not being afraid of the questions. [P. 23]*
Absence of institutional policies to integrate ACP into clinical practice	*“[…] The word cancer needs to be addressed… this disease terrifies everyone. […] So, we would need institutional support for that. Psychological support, a group, something should be done to help us deal with this topic. Because it’s a very difficult disease, and the burden it carries is immense…” [P. 32]*
Lack of training for healthcare professionals to discuss ACP with patients	*“[…] I haven’t talked to the doctor yet, I don’t know […] how to start the conversation […]. Will he know how to answer if I ask?” [P. 62]*
Biomedical model focused on disease treatment, with little integration of anticipatory care	*“[…] During the consultation, it should go like this: ‘Look, you have cancer, and these are all the possible treatment options […], which one would you choose? And if your cancer gets worse, you might face these situations, what would you prefer then?” [P. 62]*

*Notes*: *ACP: Advance Care Planning; EoL: End-of-life; P: Participant.*

**Figure 1.**
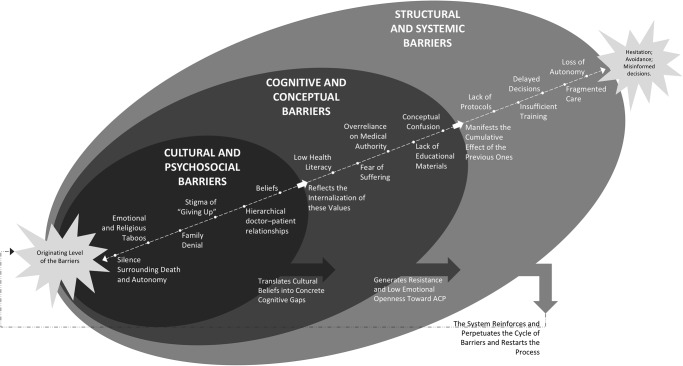
Conceptual map of the evolving pathway and dynamic interactions among barriers to Advance Care Planning.

*Notes:* *ACP: Advance Care Planning.*

Figure 1 illustrates the interaction among the main barriers to ACP. The conceptual map highlights how cultural and psychosocial, cognitive and conceptual, and structural and systemic domains overlap, reinforcing one another and shaping patients’ limited understanding and engagement with ACP.

The conceptual map demonstrates that barriers to ACP are not isolated phenomena but rather intersecting layers of cultural, cognitive, and structural factors that mutually reinforce one another. Cultural norms that discourage open dialogue about death, combined with low health literacy and limited institutional support, perpetuate silence and avoidance regarding future care.

**Figure 2.**
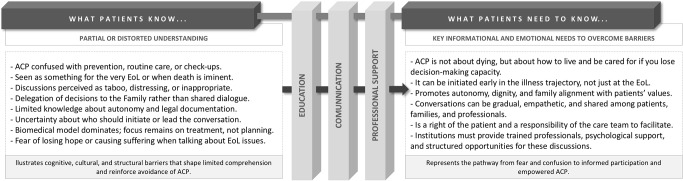
Transition from limited understanding to informed participation in Advance Care Planning: educational, communicational, and professional pillars fostering conceptual clarity and value-based engagement.

*Notes:* *ACP: Advance Care Planning; EoL: end-of-life*.

Figure 2 synthesizes what patients currently know and what they need to know, and shows the transition from patients’ partial or distorted understanding of ACP to a more informed, autonomous, and value-centered perspective.

This transition is sustained by three interconnected pillars: education, communication, and professional support, which function as bridges between misunderstanding and awareness. Education enhances conceptual clarity and health literacy; enables patients to better grasp the purpose and process of ACP. Communication fosters trust, open dialogue, and emotional safety; and professional support creating space for meaningful discussions about values and preferences. Professional support anchors the process within institutional and ethical frameworks, ensuring continuity and legitimacy in clinical practice. Collectively, these pillars promote a transformation from fear and confusion to informed engagement and empowered shared decision-making, fostering a culture of autonomy and meaningful dialogue in ACP.

## Discussion

The findings of this qualitative study highlight a significant gap in Brazilian cancer patients’ understanding of ACP. Many participants were unable to define ACP or distinguish it from general or preventive care. The analysis identified several challenges, including confusion between ACP and preventive health measures, as well as the predominant role of family members and physicians in decision-making. Emotional readiness for EoL decisions also influenced patients’ engagement with ACP (Arias-Rojas et al. 2025b). Without a clear understanding of the concept, patients may struggle to express their preferences, increasing the likelihood that EoL decisions will be made without active involvement (Mori et al. 2025). These findings raise important concerns regarding patient autonomy, shared decision-making, and the role of healthcare professionals in facilitating discussions on future care. Addressing these issues requires targeted education interventions and broader cultural shift to normalize proactive EoL conversations (Guccione et al. 2023; Paiva et al. 2023; Goswami 2024; Trevizan et al. 2024).

The primary barrier to understanding ACP was conceptual confusion, as many patients misinterpreted it as preventive care due to the ambiguity of the term “planning.” Although some participants reported familiarity with ACP, their explanations revealed only superficial understanding, likely influenced by its limited integration into healthcare contexts dominated by prevention and treatment. The prevailing biomedical focus on cure and life prolongation reinforces the misconception that ACP seeks to prevent disease rather than guide care decisions in vulnerable or EoL situations (Arias-Rojas et al. 2025b). To address this issue, recent literature suggests rebranding ACP as “Advance Care Preparation” to better convey its intent (Guccione et al. 2023; Malhotra 2024; Pimsen et al. 2025).

In Brazil, limited awareness of ACP is compounded by a paternalistic healthcare culture, where patients often delegate medical decisions to doctors and family members (Kishino et al. 2022; Rocha Tardelli et al. 2023). Similar findings have been reported among other Latin American cancer patients (Yennurajalingam et al. 2013). Fear of mortality and complex medical choices may lead many to avoid future care discussions, transferring responsibility to trusted relatives (Board et al. 2025). The strong emphasis on family involvement reinforces patients’ tendency to relinquish autonomy, often without fully understanding their rights (Kishino et al. 2022). This delegation is further fueled by misconceptions about ACP, such as the belief that planning for future care is unnecessary or carries a negative connotation (Leung et al. 2024). The lack of proactive discussions forces healthcare teams to make critical decisions under pressure, often relying on family members overwhelmed by emotional stress, uncertainty, and feelings of guilt and failure for deciding on behalf of a loved one (Kishino et al. 2022; Board et al. 2025; Pimsen et al. 2025).

Another prominent phenomenon in studies on the Brazilian population is the pervasive fear of death, including discussing it (Trevizan et al. 2024; Vidal et al. 2024). A common belief in Brazil is that “talking about death it closer,” reinforcing the fear that merely mentioning it could lead to its occurrence. Consequently, fear and psychological resistance hinder conversations about finitude and impact the acceptance of ACP in Brazil (Rocha Tardelli et al. 2023; Trevizan et al. 2024). Deeply ingrained cultural norms discourage open conversations about mortality, reinforcing avoidance that delays or prevents EoL care discussions (Zhu et al. 2025; Arias-Rojas et al. 2025b). This cultural silence is compounded by emotional barriers, as acknowledging mortality triggers anxiety, leading individuals to postpone or reject ACP as uncomfortable or unnecessary (Izumi et al. 2024). Additionally, religious and spiritual beliefs often frame death as a transcendental event beyond individual control, weakening the perception of ACP as a proactive autonomous process. As a result of these barriers, critical EoL decisions are often made in crisis situations, without the patient’s direct involvement, increasing the likelihood of misaligned interventions (Rocha Tardelli et al. 2023; Goswami 2024; Faiman 2025).

The multilevel model shows that the barriers to ACP emerge through an interconnected process shaped by sociocultural dynamics and systemic inertia. Cultural taboos surrounding death, dependence on family decisions, and the moral framing of “not giving up” act as collective defenses against existential anxiety while limiting discussions about autonomy (Pimsen et al. 2025; Arias-Rojas et al. 2025b). These cultural forces become internalized, fostering low health literacy and reinforcing beliefs that medical authority should dominate care decisions, which the healthcare system mirrors by prioritizing technical interventions over relational communication and reflective decision-making (Vanderhaeghen et al. 2020; Dias et al. 2022; Goswami 2023; Zabawa 2023; Faiman 2025; Yan et al. 2025).

Consequently, institutions become structured around procedural efficiency rather than dialogical care, producing insufficient training, lack of protocols, and fragmented coordination (Trevizan et al. 2024; Tardelli et al. 2025). This escalation reveals a feedback loop: cultural avoidance fosters cognitive confusion, which sustains structural neglect, and systemic deficiencies, in turn, validate the original cultural silence (Arias-Rojas et al. 2025b). Breaking this cycle, therefore, requires interventions that do not merely educate or legislate but that reconfigure how care, communication, and death are collectively understood within the sociocultural health systems (Faiman 2025; Yan et al. 2025).

To address these challenges, a multifaceted approach is required. First, patient and healthcare professional education is essential to clarify ACP as a patient-centered process, not preventive care (Arias-Rojas et al. 2025b; Mori et al. 2025; Pimsen et al. 2025; Volandes et al. 2025). Educational initiatives should offer accessible materials and training that enhance understanding and communication skills among both groups. Second, culturally and emotionally sensitive communication is key to reducing resistance, with providers emphasizing empathy and respecting cultural and religious views while promoting autonomy in decision-making (Izumi et al. 2024; Rak et al. 2025). Third, normalizing ACP during routine visits helps eliminate misconceptions and positions it as a standard part of quality care, not just EoL care. Additionally, shifting from therapeutic obstinacy to patient-centered care requires systemic efforts to reframe ACP as a proactive tool (Leung et al. 2024; Pimsen et al. 2025). These strategies improve autonomy, reduce family burden, and enhance care quality (Vanderhaeghen et al. 2020; Volandes et al. 2025; Arias-Rojas et al. 2025b).

This study presents some limitations. It was conducted exclusively with Brazilian women with breast cancer recruited from a single cancer center, which may limit the transferability of findings to other populations, genders, and healthcare contexts. Nonetheless, this focus enabled a deep understanding of ACP perceptions within a culturally representative and vulnerable group. The online format may have excluded participants with limited digital access, yet it facilitated geographic diversity and provided comfort for discussing sensitive topics. As with all qualitative research, results reflect subjective experiences, but this approach was crucial to capture the emotional and sociocultural meanings often overlooked in quantitative designs. Cultural and linguistic nuances may also have influenced interpretations, though they enrich comprehension of how beliefs and communication styles shape ACP engagement. Future studies should include more diverse participants, and adopt longitudinal or multicenter designs. Interventional research testing educational or communication-based strategies is also warranted to strengthen ACP literacy and inform policies that promote culturally, equitable, and patient-centered care across Latin America.

This study revealed a critical gap in Brazilian cancer patients’ the understanding of ACP. Most participants demonstrated limited or inaccurate knowledge, often confusing ACP with preventive or routine healthcare. Cultural norms that discourage discussions about death, reliance on physicians and family members for decision-making, and the absence of institutional policies further hinder ACP engagement. These findings contribute to a broader understanding of how sociocultural dynamics shape ACP engagement in collectivist healthcare contexts, highlighting the interplay between autonomy, family roles, and institutional structures. Addressing these multilevel barriers requires coordinated strategies that health literacy, promote culturally sensitive communication strategies, and build institutional support. Reframing ACP as a proactive and relational process that aligns care with patients’ values and preferences may foster earlier, more meaningful, and ethically grounded conversations about future care, ultimately enhancing the quality and dignity of EoL experiences in Brazil and similar settings.
